# Linear relationship between the Framingham Steatosis Index (FSI) and colorectal cancer (1999–2018): A nationwide cross-sectional study based on NHANES

**DOI:** 10.1097/MD.0000000000049536

**Published:** 2026-07-03

**Authors:** Binxu Wang, Bingchao Li, Long He, Cheng Xing, Bowen Sui

**Affiliations:** a Clinical Specialty in Integrative Chinese and Western Medicine, Heilongjiang University of Chinese Medicine, Harbin, Heilongjiang, China; b Department of Oncology, First Affiliated Hospital, Heilongjiang University of Chinese Medicine, Harbin, Heilongjiang, China.

**Keywords:** colorectal cancer, CRC, fatty liver disease (FLD), Framingham Steatosis Index (FSI), NHANES database

## Abstract

Colorectal cancer (CRC) poses a significant challenge to public health worldwide. Mounting evidence implicates fatty liver disease as an accelerator of CRC. This study investigated the association between the Framingham Steatosis Index (FSI), also known as the Fatty Liver Substitution Index, and CRC susceptibility. This study aims to provide a theoretical foundation for developing preventive strategies and therapeutic interventions for individuals at high risk of CRC, thereby ultimately improving treatment outcomes. This study employed 1,01,316 baseline records from the NHANES database (1999–2018). The application of stringent eligibility criteria yielded 42,732 qualified subjects: 274 individuals with CRC and 42,458 control individuals. FSI quantification was performed using standardized procedures. Following covariate adjustment, analyses were performed using multivariate logistic regression, restricted cubic spline models, and stratification approaches to ascertain the relationship between FSI scores and CRC susceptibility. Within crude models, increased FSI levels were positively associated with CRC susceptibility, maintaining this direct association after covariate adjustment. Subgroup evaluations generated uniformly consistent findings across population categories, confirming the robustness of the findings. Dose-response analyses revealed a significant linear correlation, in which an increase in FSI magnitude corresponded to a heightened CRC probability. Increasing FSI levels increase the risk of developing CRC, providing new theoretical support for the diagnosis and treatment of CRC. However, cross-sectional studies aim to establish associations, and the causal relationship between the 2 requires further research to validate and assess the feasibility of applying these findings in clinical practice.

## 1. Introduction

Recent data indicate that colorectal cancer (CRC), ranked as the third most prevalent malignancy worldwide, constitutes a primary contributor to cancer mortality, imposing a significant burden on public health.^[1]^ Advances in endoscopic screening technology have improved the detection rate of early-stage CRC. Treatment methods, such as surgery, can reduce mortality rates among early-stage patients; however, the prognosis for patients with advanced-stage disease remains poor.^[2]^ The detection of modifiable risk determinants and implementation of strategic interventions among populations at elevated risk are of critical importance. Based on previous research, the development and progression of CRC are highly complex. Key established determinants include hereditary conditions, such as familial adenomatous polyposis, obesity, inflammatory bowel disease, tobacco usage, alcohol intake, and processed meat consumption.^[3]^ Fatty liver disease (FLD) has long been overlooked and under-discussed as an emerging risk factor affecting CRC incidence.

FLD involves abnormal lipid accumulation in liver cells and progresses from early-stage steatosis to cirrhosis and advanced fibrosis. As the most prevalent hepatic disorder globally, it comprises 2 distinct subtypes: nonalcoholic fatty liver disease (NAFLD) and alcoholic fatty liver disease.^[4,5]^ Researchers believe that FLD may trigger various extrahepatic diseases, including tumor formation, potentially affecting multiple systems in the human body.^[6,7]^ FLD is a significant risk factor for CRC development and operates through mechanisms similar to those observed in diseases such as diabetes and obesity. This may be associated with metabolic disorders and systemic hyperinflammation.^[8,9]^ Research also indicates that excessive accumulation of liver fat in FLD can promote the development of CRC by disrupting the gut microbiota.^[10,11]^ Sonographic liver assessment (LUS) is the principal diagnostic method for detecting FLD. When sonographic resources are limited, researchers have developed the Framingham Steatosis Index (FSI), a composite measure incorporating hypertension, diabetes mellitus, and body mass index (BMI), as a reliable hepatic steatosis indicator. Compared with LUS, FSI provides superior accessibility, reduced costs, and broader practical implementation in various clinical contexts.^[12-14]^ Relevant studies have demonstrated that FLD promotes CRC development through multiple mechanisms; however, evidence linking the FSI to CRC risk remains insufficient.

Long et al (2016) pioneered the use of the FSI through a population-based study of 1181 subjects. Applying stepwise regression modeling, this seminal work discerned demographic profiles, clinical variables, and biochemical markers linked to hepatic steatosis, validating the FSI as a quantitative metric for liver fat accumulation.^[15]^ Motamed et al’s cohort study revealed the FSI’s diagnostic efficacy in detecting NAFLD and demonstrated robust predictive capability for new-onset NAFLD identification.^[16]^ Jung et al further evaluated hepatic steatosis using a population-based health surveillance program. Their analysis indicated that the FSI had a superior diagnostic capability relative to the Hepatic Steatosis Index and Fatty Liver Index.^[17]^ This study aimed to elucidate the multidimensional association between the FSI and cancer risk and construct conceptual frameworks for reducing CRC mortality through preventive strategies.

## 2. Materials and methods

### 2.1. Data collection and participants

This investigation utilized the NHANES database, a population study assessing health and nutritional parameters in American pediatric and adult populations. The publicly available dataset incorporates 5 key elements (demographic profiles, dietary intake documentation, clinical assessment indices, laboratory biomarkers, and questionnaire-derived responses), retrievable at https://wwwn.cdc.gov/nchs/nhanes/default.aspx. Ethical approval for this dataset was granted by the National Center for Health Statistics Institutional Review Board (Centers for Disease Control and Prevention, USA). Prior to enrollment, all subjects provided documented informed consent.^[18,19]^ This study employed NHANES records covering 1999 to 2018, including subjects aged ≥20 years who completed the colorectal cancer questionnaire and underwent interviews with physical evaluations at mobile examination units. The analytic approach applied a complex multistage probability-weighted stratified cluster sampling methodology. CRC diagnosis relied on responses in the questionnaire regarding whether participants had been informed of their diagnosis by a physician. The FSI was derived from age, sex, BMI, triglyceride levels (TG), hypertension status, diabetes diagnosis, and the ALT/AST ratio. Statistical modeling was used to evaluate FSI-CRC risk associations, accounting for age, sex, ethnicity, and additional covariates to ensure analytical robustness.

This study utilized the NHANES database population from 1999 to 2018 as the study subjects. Inclusion criteria: age ≥ 20 years; complete baseline questionnaires, physical examination records, and laboratory data; valid responses to the colorectal cancer questionnaire; availability of core data required for FSI score calculation, including age, sex, BMI, TG, hypertension, diabetes, and ALT/AST ratio; no missing data for core outcomes or exposure variables; all study participants provided informed consent.

**Figure 1.**
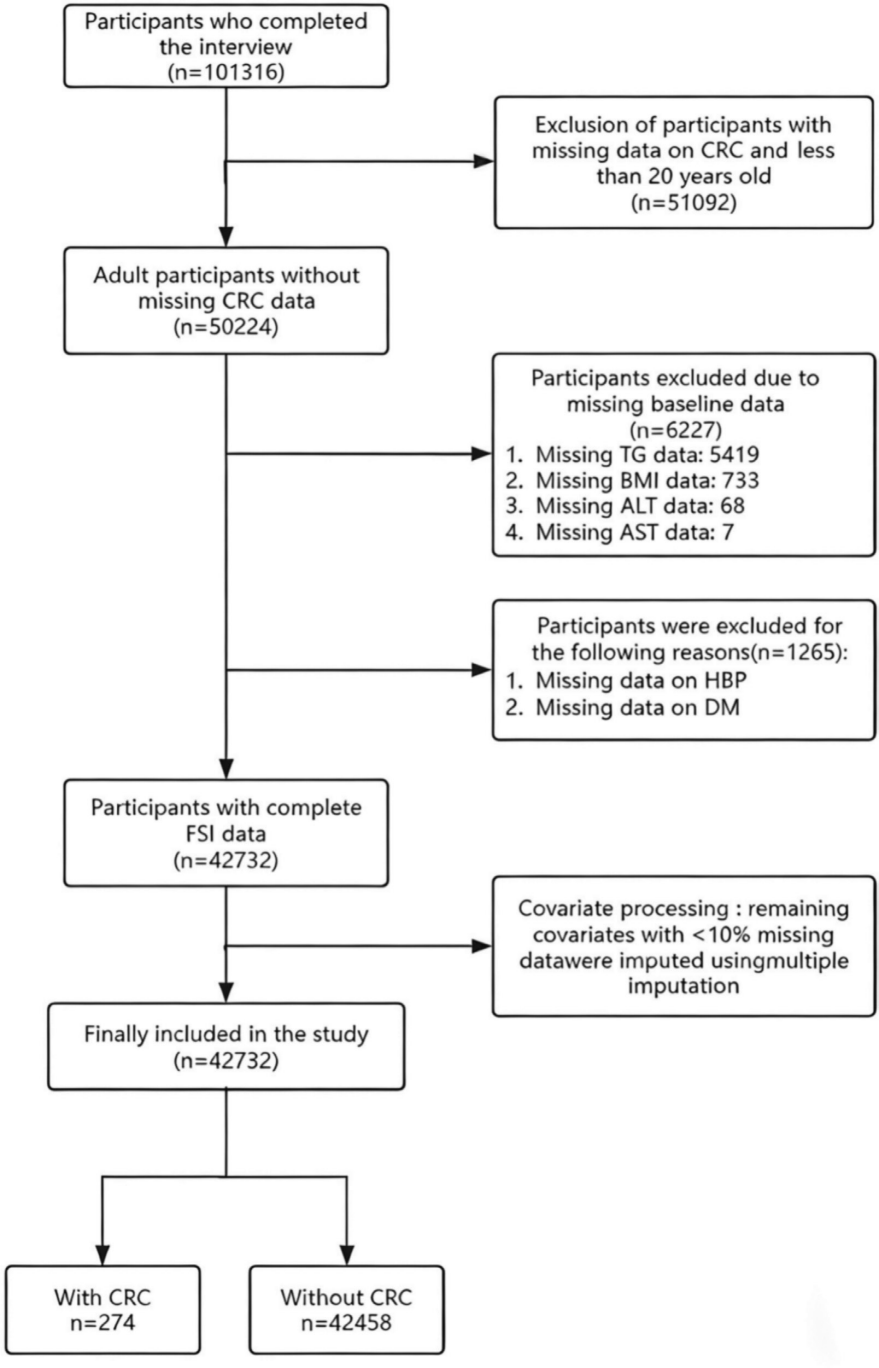
A total of 1,01,316 participants completed the interviews in this study. Among them, 51,092 participants were aged <20 years or had missing data in the CRC dataset. Subsequently, 7492 missing baseline data points were excluded from the FSI calculations. Finally, multiple imputations were performed on the remaining covariates with <10% missing data. ALT = alanine aminotransferase, AST = aspartate aminotransferase, BMI = body mass index, CRC = colorectal cancer, DM = diabetes mellitus, FSI = Framingham Steatosis Index, HBP = high blood pressure, TG = triglyceride.

Of 1,01,316 participants potentially eligible for inclusion, the following were excluded: age < 20 years and missing data in the colorectal cancer questionnaire (n = 51,092); incomplete key indicators used for FSI calculation (n = 7492); missing rate of major confounding variables ≥10% (n = 0). After exclusion based on these criteria, data from 42,732 participants were ultimately included in the final analysis. Figure 1 illustrates the complete inclusion and exclusion processes.

### 2.2. Definition and measurement of variables

#### 2.2.1. Diagnosis of CRC

Cases of CRC were verified using medical history records in the NHANES instrument. Affirmative responders to MCQ220 (“Did a physician ever diagnose you with cancer?”) We classified these individuals as having malignancies. They provided detailed information on cancer types via the Multiple Choice Questionnaire 230A (MCQ230A), with interviewers assigning specific diagnostic codes. The study cohort comprised participants with colon (coded as 16) or rectal (coded as 31) cancers. The implementation of stringent inclusion/exclusion criteria resulted in 274 pathologically validated cases of CRC.

#### 2.2.2. Calculation of FSI

The FSI calculation employs the algorithm established by Long et al as an alternative indicator for hepatic steatosis.^[15]^ The specific formula is as follows:

FSI = −7.981 + 0.011 × age (years) − 0.146 × gender (female = 1, male = 0) + 0.173 × BMI (kg/m^2^) + 0.007 × triglycerides (mg/dL) + 0.593 × hypertension (yes = 1, no = 0) + 0.789 × diabetes (yes = 1, no = 0) + 1.1 × ALT:AST ratio ≥ 1.33 (yes = 1, no = 0).

Serum alanine transaminase (ALT), aspartate transaminase (AST) and TG: Morning fasted serum specimens were acquired after ≥8 hours without food intake. TG and ALT concentrations were determined using enzymatic colorimetric assays, whereas AST levels were determined using kinetic ultraviolet analysis following standardized protocols to guarantee measurement accuracy and experimental reproducibility.^[20]^

#### 2.2.3. Selection of covariates

The results from the mobile screening center were obtained through comprehensive data collection using computer-assisted personal interviews. Demographic data included age, sex, ethnicity, educational attainment, and poverty income ratio. Lifestyle factors, such as smoking and alcohol consumption, were also considered, and the risks of hypertension, diabetes, coronary heart disease, and stroke were assessed using self-reported health data. Biochemical data included ALT, AST, blood urea nitrogen (BUN), serum creatinine (Cr), serum total cholesterol (TC), and high-density lipoprotein (HDL) cholesterol. Race was categorized as non-Hispanic White, non-Hispanic Black, Mexican American, other Hispanic, and other races. Educational attainment was categorized as <grade 9, grades 9 to 12, and >grade 12. The income-to-poverty ratio was categorized as follows: “low-income earners” (≤1.3), “middle-income earners” (1.31–3.5), and “high-income earners” (>3.5). Smoking status was categorized into 3 groups: “never” (those who have smoked < 100 cigarettes in their lifetime), “former” (those with a history of smoking but who do not currently smoke), and “current” (those with a history of smoking who still smoke at present).^[21]^ Alcohol consumption was categorized as “yes” or “no” based on participants responses to the question, “Do you drink at least 12 alcoholic drinks per year?” The determination of coronary heart disease and stroke relied on self-reported information from participants following clinical diagnosis, categorized as “yes” or “no.” Hypertension diagnosis comprised 3 components: A physical examination reveals an average systolic blood pressure ≥ 140 mm Hg or a diastolic blood pressure ≥ 90 mm Hg; a history of being informed by a physician that one has hypertension; and taking prescription medication for hypertension. Meeting any one of the above criteria constitutes hypertension. The diagnosis of diabetes comprised 5 criteria: having been informed by a physician that you have diabetes; taking hypoglycemic medication or insulin; having a glycated hemoglobin A1c (HbA1c) level ≥ 6.5%; having a fasting blood glucose level ≥ 7.0 mmol/L; and Having a random blood glucose level ≥ 11.1 mmol/L. Meeting any one of the above criteria constitutes diabetes.

### 2.3. Statistical analysis

Continuous variables are expressed as means ± standard deviation, whereas categorical variables are presented as frequency counts and proportional representations. Categorical variable distributions were analyzed using Pearson’s *χ*^2^ test, while intergroup differences among FSI strata for normally distributed variables were evaluated using 1-way analysis of variance. Univariate and multivariate binary logistic regression analyses were used to investigate FSI-CRC risk relationships, generating odds ratios (OR) with 95% confidence intervals (CI).

FSI, a continuous variable, was categorized into 4 quartiles. Covariates were initially identified through univariate analysis (*P* < .05) and subsequently evaluated for clinical relevance to determine their inclusion. Ultimately, this study constructed 3 regression models: model 1 included age, sex, and ethnicity; model 2 included model 1 and was further adjusted for education level, PIR, smoking, and alcohol consumption; and model 3 included model 2 and was further adjusted for hypertension, diabetes, coronary heart disease, stroke, ALT, AST, BUN, CR, TC, and HDL. In the adjusted model 3, we examined the dose-response relationship between FSI and CRC risk. Additionally, this study assessed potential variations in the association between FSI and CRC risk across the following variables: sex, age (<65 years, ≥65 years), smoking, alcohol consumption, hypertension, diabetes, coronary heart disease, and stroke to ensure the robustness of the findings.

All statistical analyses were performed using R Statistical Software (version 4.2.2; http://www.R-project.org, The R Foundation, Vienna, Austria) and the Free Statistics analysis platform (version 2.3; http://www.clinicalscientists.cn/freestatistics, Beijing, China). All statistical results were considered statistically significant at *P* < .05.

## 3. Results

### 3.1. Study cohort characteristics

A total of 1,01,316 participants were interviewed for this study. Among them, 51,092 participants aged <20 years and those with missing CRC data were excluded. Additionally, 7492 participants were excluded due to missing baseline data used for the FSI calculation, hypertension, and diabetes. Since the remaining covariates had <10% missing data, multiple imputation was employed to fill in the gaps. After inclusion and exclusion, the study included 42,732 participants, comprising 274 patients with CRC and 42,458 non-CRC patients. Figure 1 depicts the algorithmically structured participant selection protocol according to the eligibility criteria in this cohort study.

### 3.2. Population characteristics stratified by FSI quartile

Table 1 presents the baseline attributes of 42,732 enrolled individuals stratified by FSI quartiles. Statistically significant differences (*P* < .05) emerged across several parameters, with a mean participant age of 48.2 ± 17.7 years. The uppermost FSI quartile predominantly featured males, non-Hispanic White racial identification, and advanced educational qualifications. Concurrently, CAD, cerebrovascular events, hypertensive disorders, and diabetic conditions demonstrated positive correlations with increasing FSI values.

**Table 1:** Population characteristics assessed using the Framingham Steatosis Index.

Variables	Total (n = 42,732)	1 (n = 10,683)	2 (n = 10,683)	3 (n = 10,682)	4 (n = 10,684)	*P*
Gender, n (%)						<.001
Male	20,698 (48.4)	4261 (39.9)	5288 (49.5)	5489 (51.4)	5660 (53)	
Female	22,034 (51.6)	6422 (60.1)	5395 (50.5)	5193 (48.6)	5024 (47)	
Age (yr)	48.2 ± 17.7	38.4 ± 15.5	49.4 ± 17.9	52.5 ± 17.4	52.4 ± 15.8	<.001
Education level, n (%)						<.001
<9th grade	5159 (12.1)	711 (6.7)	1312 (12.3)	1565 (14.7)	1571 (14.7)	
9th–12th grade	16,288 (38.1)	3733 (34.9)	3952 (37)	4261 (39.9)	4342 (40.6)	
>12th grade	21,285 (49.8)	6239 (58.4)	5419 (50.7)	4856 (45.5)	4771 (44.7)	
Race, n (%)						<.001
Non-Hispanic White	18,098 (42.4)	4852 (45.4)	4631 (43.3)	4270 (40)	4345 (40.7)	
Non-Hispanic Black	8920 (20.9)	2126 (19.9)	2230 (20.9)	2292 (21.5)	2272 (21.3)	
Mexican American	7973 (18.7)	1471 (13.8)	1880 (17.6)	2259 (21.1)	2363 (22.1)	
Other race	7741 (18.1)	2234 (20.9)	1942 (18.2)	1861 (17.4)	1704 (15.9)	
PIR, n (%)						<.001
Low income: ≤1.3	13,777 (32.2)	3306 (30.9)	3305 (30.9)	3352 (31.4)	3814 (35.7)	
Medium income: 1.31–3.50	16,144 (37.8)	3840 (35.9)	3992 (37.4)	4221 (39.5)	4091 (38.3)	
High income: >3.50	12,811 (30.0)	3537 (33.1)	3386 (31.7)	3109 (29.1)	2779 (26)	
Coronary heart disease, n (%)						<.001
No	41,157 (96.3)	10,589 (99.1)	10,341 (96.8)	10,165 (95.2)	10,062 (94.2)	
Yes	1575 (3.7)	94 (0.9)	342 (3.2)	517 (4.8)	622 (5.8)	
Stroke, n (%)						<.001
No	41,364 (96.8)	10,577 (99)	10,353 (96.9)	10,276 (96.2)	10,158 (95.1)	
Yes	1368 (3.2)	106 (1)	330 (3.1)	406 (3.8)		
Smoke, n (%)						<.001
Never	23,568 (55.2)	6352 (59.5)	5871 (55)	5829 (54.6)	5516 (51.6)	
Former	10,104 (23.6)	1599 (15)	2527 (23.7)	2865 (26.8)	3113 (29.1)	
Current	9060 (21.2)	2732 (25.6)	2285 (21.4)	1988 (18.6)	2055 (19.2)	
Drink, n (%)						<.001
No	11,735 (27.5)	2674 (25)	2849 (26.7)	3020 (28.3)	3192 (29.9)	
Yes	30,997 (72.5)	8009 (75)	7834 (73.3)	7662 (71.7)	7492 (70.1)	
Hypertension, n (%)						<.001
No	25,298 (59.2)	9651 (90.3)	6998 (65.5)	5205 (48.7)	3444 (32.2)	
Yes	17,434 (40.8)	1032 (9.7)	3685 (34.5)	5477 (51.3)	7240 (67.8)	
Diabetes, n (%)						<.001
No	35,971 (84.2)	10,609 (99.3)	10,154 (95)	8881 (83.1)	6327 (59.2)	
Yes	6761 (15.8)	74 (0.7)	529 (5)	1801 (16.9)	4357 (40.8)	
Laboratory metrics						
ALT (U/L)	21.0 (16.0, 28.0)	17.0 (14.0, 22.0)	20.0 (16.0, 26.0)	22.0 (17.0, 30.0)	25.0 (18.0, 37.0)	<.001
AST (U/L)	22.0 (19.0, 27.0)	21.0 (18.0, 25.0)	22.0 (19.0, 27.0)	23.0 (19.0, 28.0)	23.0 (19.0, 29.0)	<.001
BUN (mmol/L)	4.8 ± 2.1	4.3 ± 1.6	4.7 ± 1.9	5.0 ± 2.2	5.2 ± 2.5	<.001
CR (umol/L)	11,241.3 ± 7210.9	10,909.5 ± 7510.3	10,994.8 ± 7198.1	11,207.1 ± 7039.5	11,853.6 ± 7048.6	<.001
TC (mmol/L)	5.1 ± 1.1	4.7 ± 1.0	5.1 ± 1.1	5.2 ± 1.1	5.2 ± 1.2	<.001
HDL (mmol/L)	1.4 ± 0.4	1.6 ± 0.4	1.4 ± 0.4	1.3 ± 0.4	1.1 ± 0.3	<.001

ALT = alanine aminotransferase, AST = aspartate aminotransferase, BUN = blood urea nitrogen, CR = creatinine, HDL = high-density lipoprotein, TC = serum total cholesterol.

### 3.3. Association between FSI and CRC

As shown in Table 2, a univariate logistic regression analysis revealed that age, education level, ethnicity, smoking status, coronary heart disease, stroke, hypertension, diabetes, ALT level, BUN level, and CR were associated with CRC.

**Table 2:** Association between colorectal cancer and covariates.

Variable	OR (95% CI)	*P*
Gender, n (%)		
Male	1 (Reference)	
Female	0.85 (0.67–1.07)	.172
Age (yr)	1.1 (1.09–1.11)	<.001
Education level, n (%)		
<9th grade	1 (Reference)	
9th–12th grade	0.82 (0.57–1.16)	.261
>12th grade	0.67 (0.48–0.96)	.027
Race, n (%)		
Non-Hispanic White	1 (Reference)	
Non-Hispanic Black	0.56 (0.41–0.77)	<.001
Mexican American	0.25 (0.16–0.4)	<.001
Other race	0.32 (0.21–0.49)	<.001
PIR, n (%)		
Low income: ≤1.3	1 (Reference)	
Medium income: 1.31–3.50	1.13 (0.85–1.49)	.403
High income: >3.50	0.85 (0.62–1.17)	.328
Coronary heart disease, n (%)		
No	1 (Reference)	
Yes	4.42 (3.14–6.23)	<.001
Stroke, n (%)		
No	1 (Reference)	
Yes	3.35 (2.24–5.01)	<.001
Drink, n (%)		
No	1 (Reference)	
Yes	0.92 (0.71–1.19)	.519
Smoke, n (%)		
Never	1 (Reference)	
Former	2.68 (2.08–3.46)	<.001
Current	0.74 (0.5–1.09)	.126
Hypertension, n (%)		
No	1 (Reference)	
Yes	5.01 (3.77–6.65)	<.001
Diabetes, n (%)		
No	1 (Reference)	
Yes	2.29 (1.76–2.97)	<.001
ALT (U/L)	0.98 (0.97–0.99)	<.001
AST (U/L)	1 (0.98–1.01)	.375
BUN (mmol/L)	1.16 (1.13–1.19)	<.001
CR (umol/L)	1 (1–1)	.001
TC (mmol/L)	0.92 (0.82–1.03)	.149
HDL (mmol/L)	1.18 (0.9–1.56)	.231

ALT = alanine aminotransferase, AST = aspartate aminotransferase, BUN = blood urea nitrogen, CI = confidence interval, CR = creatinine, HDL = high-density lipoprotein, OR = odds ratio, TC = serum total cholesterol.

As shown in Table 3, a multivariate logistic regression analysis was conducted to assess the association between FSI and CRC risk, incorporating both adjusted and unadjusted factors. In the unadjusted model of the multivariate logistic regression analysis, FSI as a continuous variable (per 1-unit increase) was positively associated with the probability of CRC occurrence (OR, 1.13; CI, 1.08–1.18; *P* < .001). In model 1, which was adjusted for confounding factors, including sex, age, and ethnicity, the results showed a positive correlation similar to that in the unadjusted model (OR, 1.11; CI, 1.04–1.18; *P* = .002). In model 2, which was further adjusted for educational attainment, PIR, smoking, and alcohol consumption, based on model 1, the results continued to show a positive association (OR, 1.1; CI, 1.03–1.18; *P* = .004). In model 3, which was further adjusted for coronary heart disease, stroke, hypertension, diabetes, ALT, AST, BUN, CR, TC, and HDL, based on model 2, the results showed that the positive correlation remained significant (OR, 1.11; CI, 1.01–1.22; *P* = .024). After performing a quartile analysis of the FSI, the probability of subjects developing CRC increased with rising FSI levels in the unadjusted model. In the adjusted model, the probability of developing CRC continued to show an upward trend. In model 3, adjusted for all confounding factors, FSI showed an overall positive correlation with CRC prevalence. Compared with the *Q*1 group with lower FSI values, the OR for CRC prevalence in the *Q*2 group was 1.81 (CI, 1.06–3.1, *P* = .029), and the OR in the *Q*3 group was 1.72 (CI, 0.98–3.02, *P* = .059), and the OR for the *Q*4 group was 2.26 (CI, 1.22–4.17, *P* = .009). Despite negative outcomes in the *Q*3 group, the overall pooled data still suggest a positive correlation between FSI and CRC incidence. The *P*-values for trend tests were <.001, .004, .005, and .036, respectively.

**Table 3:** Association between Framingham Steatosis Index and colorectal cancer incidence.

Variables	Nonadjusted model		Model 1		Model 2		Model 3	
	OR (95% CI)	*P*	OR (95% CI)	*P*	OR (95% CI)	*P*	OR (95% CI)	*P*
FSI	1.13 (1.08–1.18)	<.001	1.11 (1.04–1.18)	.002	1.1 (1.03–1.18)	.004	1.11 (1.01–1.22)	.024
Subgroups								
*Q*1	1 (Ref)		1 (Ref)		1 (Ref)		1 (Ref)	
*Q*2	4.47 (2.68–7.46)	<.001	1.83 (1.09–3.08)	.022	1.82 (1.08–3.05)	.025	1.81 (1.06–3.1)	.029
*Q*3	4.7 (2.82–7.82)	<.001	1.75 (1.04–2.94)	.034	1.73 (1.03–2.9)	.039	1.72 (0.98–3.02)	.059
*Q*4	5.15 (3.1–8.53)	<.001	2.3 (1.38–3.83)	.001	2.25 (1.34–3.75)	.002	2.26 (1.22–4.17)	.009
Trend test	1.41 (1.26–1.58)	<.001	1.21 (1.06–1.37)	.004	1.2 (1.06–1.36)	.005	1.2 (1.01–1.42)	.036

Model 1 accounted for gender, age, and race.

Model 2 included the covariates from model 1and additionally adjusted for PIR, educational level, smoking status, and drinking status.

Model 3 incorporated all variables in model 2 and further controlled for coronary heart disease, stroke, hypertension, diabetes, ALT, AST, BUN, CR, TC, and HDL.

ALT = alanine aminotransferase, AST = aspartate aminotransferase, BUN = blood urea nitrogen, CR = creatinine, CI = confidence interval, FSI = Framingham Steatosis Index, HDL = high-density lipoprotein, OR = odds ratio, TC = serum total cholesterol.

### 3.4. The linear relationship between FSI and CRC risk

**Figure 2.**
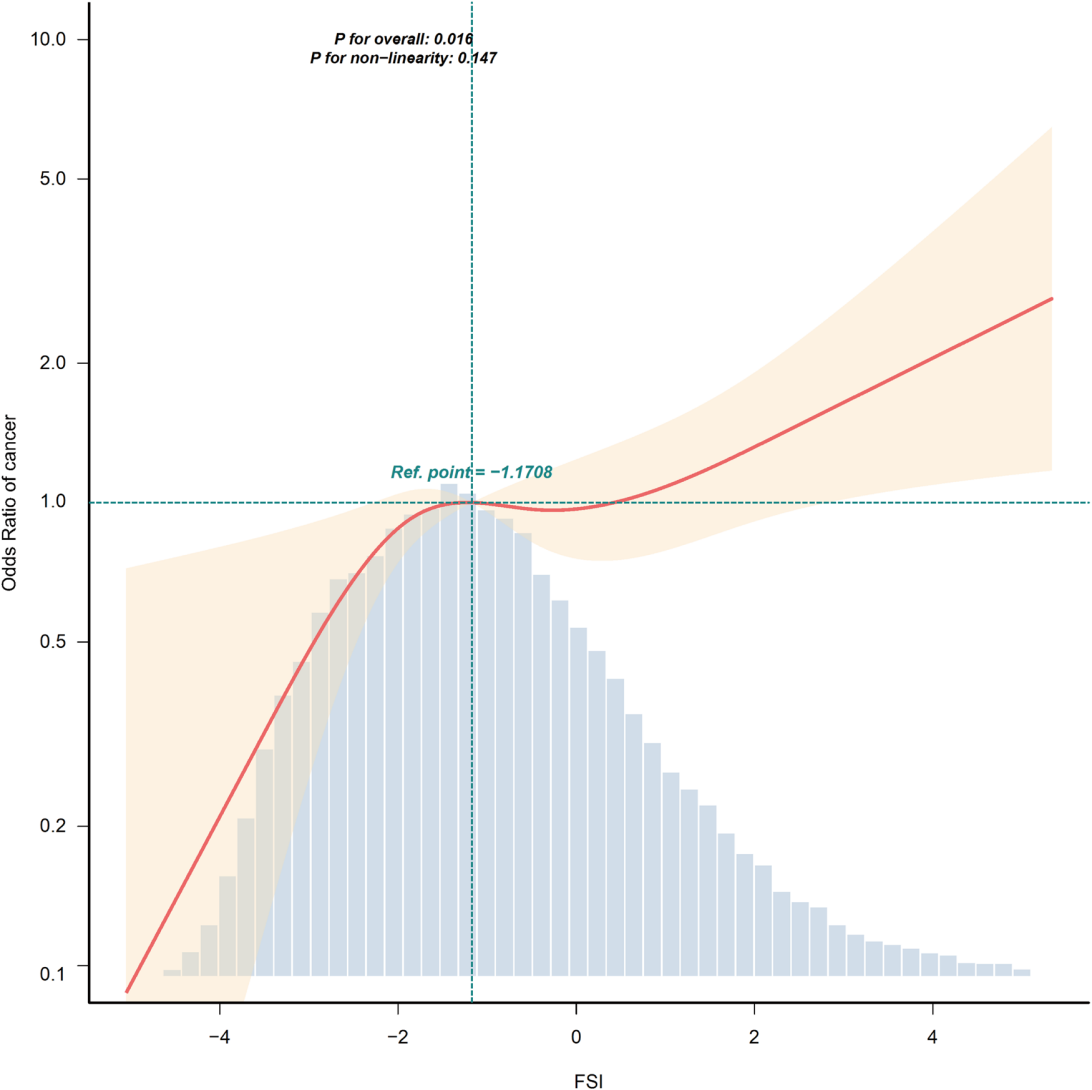
Association between FSI and CRC risk. The solid red line represents the effect size of CRC at different FSI levels, with the shaded areas indicating 95% confidence intervals. This result was adjusted for sex, age, race, education level, PIR, smoking, alcohol consumption, coronary heart disease, stroke, hypertension, diabetes, ALT, AST, BUN, CR, TC, and HDL. ALT = alanine aminotransferase, AST = aspartate aminotransferase, BUN = blood urea nitrogen, CR = creatinine, CRC = colorectal cancer, FSI = Framingham Steatosis Index, HDL = high-density lipoprotein, TC = serum total cholesterol.

A significant linear dose-response relationship linking FSI levels to CRC risk is shown in Figure 2 (*P* for non-linearity = .147). This association was determined through restricted cubic spline analysis, incorporating multivariate adjustments and exclusion of FSI outliers (upper and lower 0.5%). Overall, the results imply that the risk increases progressively with higher FSI levels.

### 3.5. Subgroup analysis of the relationship between FSI and CRC

**Figure 3.**
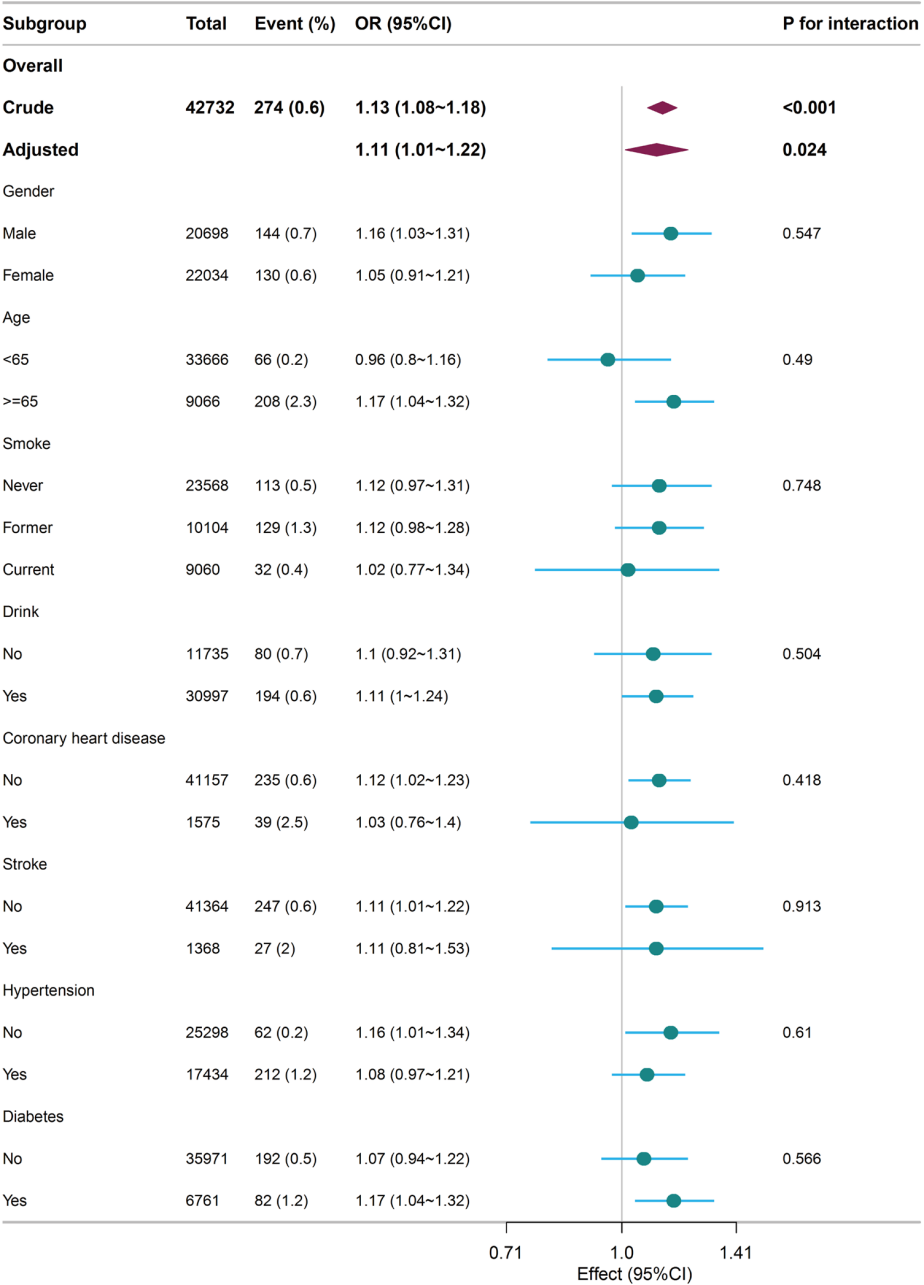
The impact of FSI on CRC risk was assessed, with results presented as adjusted FSI OR (95% CI), accounting for confounders not included in the stratified analyses. CI = confidence interval, CRC = colorectal cancer, FSI = Framingham Steatosis Index, OR = odds ratio.

As shown in Figure 3, a stratified analysis was conducted within subgroups to assess potential interactions between FSI and CRC risk. Stratification by sex, age, smoking, alcohol consumption, coronary heart disease, stroke, hypertension, and diabetes revealed no significant subgroup interactions, indicating robust subgroup analysis results.

## 4. Discussion

This cross-sectional study, which represented the entire US population, evaluated the association between FSI and CRC risk in 274 patients with CRC from the NHANES database spanning 1999 to 2018. After adjusting for a series of potential confounding factors, multivariate logistic regression analysis revealed a significant positive correlation between FSI and CRC risk. Analysis of the dose-response curve revealed a linear positive correlation between FSI and CRC risk. We also conducted subgroup analyses and found no specific subgroups, indicating that the study results were robust. To our knowledge, this study is the first to link FSI with CRC risk, aiming to explore the relationship between the 2 in depth, making it innovative.

FLD and NAFLD are systemic diseases that can affect human metabolism and inflammatory responses. Increasing research indicates a close association between FLD and CRC, as well as other malignant tumors.^[22]^ A meta-analysis of 11 observational studies demonstrated that FLD promotes the formation of colorectal adenomas and CRC, with hazard ratios (HR) and 95% CI of (1.42, [1.118–1.72]) and (3.08, [1.02–9.03]), respectively.^[23]^ Research indicates that the potential mechanisms by which FLD promotes CRC formation may involve metabolic disorders, increased inflammation, and dysbiosis of the gut microbiota.^[24,25]^

FLD represents the hepatic manifestation of metabolic syndrome, the characteristics of which – dyslipidemia, insulin resistance (IR), and obesity – constitute risk factors for developing CRC.^[26,27]^ A multicenter cohort study in Italy involving 34,148 participants observed that the risk of CRC increases with elevated levels of TC and low-density lipoprotein, with this association being more pronounced in men and postmenopausal women.^[28]^ A meta-analysis involving 19,87,753 individuals identified 10,876 cases of CRC, indicating that higher concentrations of TG and TC are associated with an increased risk of developing this malignancy.^[29]^ Dyslipidemia associated with FLD can lead to the onset of CRC, with the underlying mechanism involving increased leptin and decreased adiponectin levels. An observational study involving 32 subjects revealed through comprehensive analysis that patients with CRC exhibited systemic reductions in adiponectin levels compared to healthy controls.^[30]^ Studies have indicated that AMP-activated protein kinase facilitates the inhibition of tumor cell growth by adiponectin. This hormone promotes apoptosis induced by asparaginase, producing an anticancer effect.^[31]^ Current data indicate that administration of statins elevates leptin and adiponectin levels in FLD treatment, potentially offering a mechanism contributing to preventing CRC.^[32]^ The accumulation of fat in the liver alters insulin signaling pathways by stimulating insulin to reduce glucose uptake, thereby inducing IR.^[33]^ The insulin-like growth factor-1 axis and hyperinsulinemia represent the most reliable mechanisms linking FLD-associated IR to CRC development, creating conditions conducive to CRC onset through their antiapoptotic and proliferative effects.^[34]^ In a prospective cohort study targeting Chinese adults, the results indicated that an elevated fasting triglyceride-glucose index (TyG; another indicator of IR) is demonstrated to correlate with a heightened probability of CRC.^[35]^ FLD and CRC are both influenced by obesity factors.^[10]^ Although this conclusion lacks strong theoretical support in our current research, obese patients are often advised to undergo colonoscopy at an early stage. Current research has attested to FLD occurring in nonobese patients, opposing the established obesity-FLD association. Studies have demonstrated that lean NAFLD cases present characteristic metabolic alterations, enhancing susceptibility to CRC irrespective of BMI.^[36]^ Current findings demonstrate the complex interplay within human metabolic systems and the varied expression of CRC risk factors across individual cohorts. Specifically, distinct biological mechanisms underlie the malignant transformation of overweight and lean individuals. Supporting these data, determinants unrelated to obesity have been found to correlate with FLD development and substantially influence CRC progression.

Chronic inflammation in FLD may be a plausible pathogenic mechanism underlying CRC development. Key mediators, such as tumor necrosis factor-α, interleukin-6, and plasminogen activator inhibitor-1, foster tumor growth by enhancing cell division, blocking apoptosis, and disrupting blood vessel formation. Collectively, these actions accelerate CRC pathogenesis.^[37,38]^ A chronic low-grade inflammatory state in visceral adipose tissue is identified as an independent predictor of FLD progression. The adipokines released from this tissue induce IR in the body while simultaneously promoting the development of CRC.^[39]^ Additionally, FLD induces inflammation during its pathogenic process, which subsequently alters gut microbiota – a process closely associated with the development of CRC.^[40,41]^ Catalyzed by disrupted bacterial metabolites, Toll-like receptors are activated by disrupted bacterial metabolites, which may represent the underlying mechanism of these hypotheses.^[42]^ However, the underlying mechanisms of these associations require further research to substantiate them.

## 5. Limitations

This study has several methodological constraints. Initially, a detailed analysis stratified by CRC stage was omitted, preventing sensitivity assessments related to disease progression. Subsequently, as data on patient survival and therapeutic results were unavailable, the impact on quality of life could not be evaluated. Furthermore, the study cohort was predominantly US residents; consequently, generalizing these results to other geographical areas, such as Asia, necessitates further investigation. Finally, the identification of CRC cases relied on self-administered questionnaires, potentially missing some diagnoses.

## 6. Conclusion

This study utilized the NHANES database to demonstrate that the FLD substitution index (FSI) was positively correlated with CRC risk without specific population exceptions, indicating the robustness of our findings. FSI serves as a convenient non‑invasive marker for CRC risk stratification, offering new epidemiological evidence connecting hepatic steatosis to colorectal carcinogenesis.

Given the cross‑sectional design, causality cannot be established. Large prospective cohort studies are needed to verify the temporal association between FSI and incident CRC, particularly in Asian populations to enhance generalizability. Further research should clarify the molecular mechanisms of FSI‑related metabolic disorders, inflammation and gut microbiota dysregulation in CRC development. Long‑term studies are also required to evaluate FSI‑guided screening and intervention for hepatic steatosis in reducing advanced colorectal neoplasia risk and improving prognosis.

## Author contributions

**Data curation:** Binxu Wang, Bingchao Li.

**Formal analysis:** Binxu Wang, Long He.

**Investigation:** Binxu Wang, Bingchao Li.

**Methodology:** Long He, Cheng Xing.

**Project administration:** Binxu Wang.

**Software:** Binxu Wang, Bingchao Li, Long He.

**Supervision:** Bowen Sui.

**Validation:** Binxu Wang, Bingchao Li.

**Visualization:** Cheng Xing.

**Writing – original draft:** Binxu Wang.

**Writing – review & editing:** Bowen Sui.
